# Lexical decisions in adults with low and high susceptibility to pattern-related visual stress: a preliminary investigation

**DOI:** 10.3389/fpsyg.2015.00449

**Published:** 2015-04-14

**Authors:** James M. Gilchrist, Peter M. Allen

**Affiliations:** ^1^School of Optometry and Vision Science, University of BradfordBradford, UK; ^2^Vision and Eye Research Unit, Department of Vision and Hearing Sciences, Anglia Ruskin UniversityCambridge, UK

**Keywords:** vision, reading, visual stress, word recognition, lexical decision

## Abstract

Pattern-related visual stress (PRVS) is a form of sensory hypersensitivity that some people experience when viewing high contrast repeating patterns, notably alternating dark and light stripes. Those susceptible to PRVS typically have a strong aversion to such stimuli, and this is often accompanied by experiences of visual discomfort and disturbance. The patterns most likely to elicit symptoms of PRVS have a square-wave grating configuration of spatial frequency ~3 cycles/degree. Such stimuli are characteristic of printed text in which lines of words and the spaces between them present a high contrast grating-like stimulus. Consequently, much printed reading material has the potential to elicit PRVS that may impair reading performance, and this problem appears to be common in individuals with reading difficulties including dyslexia. However, the manner in which PRVS affects reading ability is unknown. One possibility is that the early sensory visual stress may interfere with the later cognitive word recognition stage of the reading process, resulting in reading performance that is slower and/or less accurate. To explore the association of PRVS with word recognition ability, lexical decision performance (speed and accuracy) to words and pronounceable non-words was measured in two groups of adults, having low and high susceptibility to PRVS. Results showed that lexical decisions were generally faster but less accurate in high-PRVS, and also that high-PRVS participants made decisions significantly faster for words than for non-words, revealing a strong lexicality effect that was not present in low-PRVS. These findings are novel and, as yet, unconfirmed by other studies.

## Introduction

Reading is a complex and demanding activity, requiring efficient processing and integration of visual, phonological and semantic information with eye movement control and coordination. Research on the reading process, and on factors that impair reading ability, is already extensive but some issues remain unexplored and unexplained. This article addresses an aspect of one such issue, how visual word recognition may be affected by a commonly encountered sensory phenomenon called pattern-related visual stress (PRVS). To provide a context for considering this question we will first summarize the stages of the reading process and later describe the nature of PRVS.

### The reading process

The process of reading text may be conceived as follows: (1) the reader selects and fixes on a word, (2) the word (or a group of words around fixation—the “perceptual span”) is decoded to determine whether it can be identified and recognized as meaningful, then the decoded word/span representation is conveyed to working memory pending the decoding of further words/spans, (3) saccadic eye movements bring about a change in fixation to select the next word/group of interest, then steps (1)–(3) are repeated. Through this process of repeated selection, decoding and saccade/fixation an interpretation of the meaning of the text is constructed in working memory. In spite of its simplicity, this intuitive 3-stage description captures the essence of some influential models of reading that recognize the need to combine word recognition with visual attention and eye movements to create fluency for meaningful interpretation of text (e.g., Reichle et al., 2003; Engbert et al., 2005; Rayner and Reichle, 2010).

The first stage of the reading process is *visual sensory/perceptual* and is influenced by stimulus parameters such as print size (Chung et al., 1998; Legge and Bigelow, 2011), contrast (Legge et al., 1987), and blur (Legge et al., 1985; Chung et al., 2007), and also by perceptual factors such as the visual span (Legge et al., 2007), and crowding (Pelli et al., 2007).

The second, word recognition, stage in reading is *cognitive* and includes visual feature integration, orthographic processing (Humphreys et al., 1990; Grainger and Jacobs, 1996), phonological coding (Pollatsek et al., 2000), and semantic processing (Price et al., 1997; Stolz and Besner, 1998; Rastle et al., 2000). It is widely accepted that the process of word recognition incorporates the three elements mentioned above: orthographic processing of the visual content of a word, phonological processing of the word sound, and semantic processing to deliver its meaning (e.g., Seidenberg and McClelland, 1989). The word recognition process may be subdivided into auditory and visual aspects relating, respectively, to recognition of words that are heard and seen. Semantic interpretation of spoken words may be achieved by direct auditory/phonological analysis without the need for orthographic processing while, similarly, semantic interpretation of printed words may be achieved by direct visual/orthographic analysis without the need for phonological processing (e.g., Coltheart, 2004). Visual word recognition, which may be thought of as the cognitive foundation for reading, has been studied extensively and a number of computational models developed (Norris, 2013). In addition, neurophysiological methods have been used to complement computational and behavioral approaches (Carreiras et al., 2014).

The third stage of the reading process is *oculomotor*, involving programming and execution of saccades followed by steady fixation (Rayner, 1998, 2009). Across all 3 stages of the process described above, there is also important involvement of visual attention, both in control of saccadic eye movements (Hoffman and Subramaniam, 1995; Inhoff et al., 2000) and in word recognition (Pammer et al., 2006; Lobier et al., 2012).

The present study examines a specific aspect of the interaction between stages 1 and 2 of this conceptual model of reading; that is the possible effect of the visual sensory phenomenon known as pattern-related visual stress on the cognitive process of word recognition as represented by performance on a visual lexical decision task.

### Pattern-related visual stress (PRVS)

Pattern-related visual stress is a visual sensory hypersensitivity that some people experience when viewing high contrast repeating patterns, notably alternating dark and light stripes. Individuals susceptible to PRVS typically have a strong aversion to viewing such stimuli, and this is often accompanied by headaches, eyestrain, sensations of excessive brightness or contrast, visual disturbance (fading, blurring, flickering and movement of parts of the stimulus), and occasionally by vertigo and nausea (Wilkins et al., 1984; Wilkins, 1995).

The condition, which here we call PRVS, has also been called *visual discomfort* (Conlon et al., 1999, 2001; Borsting et al., 2007), *scotopic sensitivity syndrome* (Irlen, 1994), *Irlen syndrome* (Irlen, 1994), and *Meares-Irlen syndrome* (Evans et al., 1996). Here we favor the term pattern-related visual stress (PRVS) as the condition not only appears to be specifically provoked or exacerbated by patterned stimuli but also because it may be characterized by visual disturbance as well as discomfort, and because the “syndrome” terminology provides no descriptive insight into the condition and is therefore unhelpful. In particular, *scotopic sensitivity* is inappropriate and misleading as the condition is not typically associated with scotopic vision. Therefore, use of the terms *scotopic sensitivity* and *(Meares-)Irlen syndrome* in this context are deprecated.

Occurrence of PRVS has been reported in individuals who suffer from migraine (Marcus and Soso, 1989; Harle et al., 2006), photosensitive epilepsy (Wilkins et al., 1979, 1980), in cases of stroke (Beasley and Davies, 2012) and in Chronic Fatigue Syndrome (Loew et al., 2014). Individuals susceptible to PRVS may also experience impaired visual-search performance (Conlon et al., 1998; Conlon and Humphreys, 2001; Allen et al., 2008).

The stimulus characteristics most likely to elicit symptoms of PRVS are described by Wilkins (1995; see also Conlon et al., 2001). Patterns of high contrast having a striped “grating” configuration of spatial frequency around 3 cycles/degree, and with stripes of equal width and spacing (duty cycle of approximately 50%) tend to produce maximum effect in PRVS-susceptible individuals. The capacity of such patterns to provoke visual discomfort and disturbance has been confirmed in recent studies by Fernandez and Wilkins (2008), Juricevic et al. (2010) and O'Hare and Hibbard (2011). These studies are in broad agreement that visual stress is particularly associated with stimuli whose spatial statistics deviate from those of natural images, and one possibility is that such patterns have a general tendency to cause discomfort because the visual system is optimized for viewing natural scenes in which regular, high-contrast patterns tend to occur infrequently.

As noted above, high-contrast regular patterns likely to provoke PRVS occur more frequently in man-made environments. Perhaps their most commonplace occurrence is in printed text, where lines of text and the spaces between them present a grating-like stimulus. When text is printed in black against a white background, as is most often the case, the pattern also exhibits high contrast. Consequently, much printed reading material has the potential to elicit PRVS in certain individuals (Meares, 1980; Wilkins and Nimmo-Smith, 1987; Wilkins, 1993; Irlen, 1994).

In relation to its effect on reading, some reports have suggested that PRVS is particularly prevalent in individuals having dyslexia (Kriss and Evans, 2005; Singleton and Trotter, 2005). For other authors, the existence itself of PRVS as an independent condition affecting reading ability is controversial (Henderson et al., 2014; Uccula et al., 2014). However, recent work using both fMRI (Huang et al., 2011) and near-infrared spectroscopy (Coutts et al., 2012) lend significant support for its existence, and its possible association with reading difficulty/dyslexia continues to be of interest to many researchers (Conlon, 2012; Singleton, 2012).

In fact the level of interest in, and controversy around, the possible association between visual stress and dyslexia was the primary motivation for the present study. The manner in which PRVS may affect reading ability remains unclear. Here we consider a possible factor, which is that the visual sensory experience of PRVS might interfere with the cognitive process of visual word recognition as measured by performance on a visual lexical decision task.

### Visual lexical decision (VLD)

The visual lexical decision task requires participants to decide whether a stimulus that is presented is a real word or a fictitious non-word. Performance on the lexical decision task is measured in terms of response time and word/non-word recognition accuracy. Theoretically the lexical decision task requires rapid retrieval of words from lexical memory (Harm and Seidenberg, 2004). In the case of a non-word the participant must perform an exhaustive search of the lexicon before they can accurately reject the stimulus as a non-word. It has been shown (Katz et al., 2012) that performance on the VLD task predicts word identification ability as measured with the Test of Word Reading Efficiency (TOWRE; Torgesen et al., 1999). Thus, VLD appears to provide an effective paradigm for inferences on individual differences in word recognition.

Use of lexical decision to study performance in visual word recognition offers a distinct advantage over the most common alternative approach of word naming, in that the latter typically requires the involvement of phonological processing for naming of low frequency words and non-words, which cannot be recognized immediately by sight but must be “sounded out” before they can be identified and named (Coltheart et al., 2001). Thus, word naming requires use of phonology to perform the task, but lexical decision does not (Coltheart et al., 1979).

The aim of the present study is to investigate whether two participant groups, having low and high susceptibility to pattern-related visual stress, differ in their abilities to discriminate between words and (pronounceable) non-words in a visual lexical decision task. Previous studies have examined the influence of visual stimulus quality on word recognition, particularly in relation to the effect of word frequency (e.g., Yap et al., 2008) but the current study does not involve direct degradation of the visual stimulus. We are not aware of any other study investigating the association between visual discomfort/visual stress and word recognition.

Our hypothesis may be stated in terms of the claims, described above, that pattern-related stress is often associated with impaired reading performance. If PRVS impairs word recognition ability, and VLD provides an indication of the latter, then we might expect participants with high-PRVS to make lexical decisions more slowly and/or less accurately than those with low-PRVS. The following experiment tests this hypothesis.

## Materials and methods

### Ethics statement

The investigation adhered to the principles of the Declaration of Helsinki, and was approved by a local internal ethics committee at Anglia Ruskin University where the data were collected. Informed oral consent was obtained from every participant after a verbal and a written explanation of the procedures was given.

### Participants

Twenty eight participants were recruited, 12 male and 16 female aged from 18 to 65 years (overall mean 38.0, sd 11.0). All had normal or corrected-to-normal vision; could read N5 at 0.4 m and had distance visual acuity of at least -0.1 logMAR. None of the participants had ever been classified as having dyslexia or as having a reading disability, and none had ever been treated for any binocular or oculomotor anomaly. All had amplitudes of accommodation that were normal for age and normal binocular convergence and ocular motility. Other optometric data were not obtained because previous studies have suggested that subtle binocular and accommodative anomalies are not major aetiological factors in visual stress (Evans et al., 1995, 1996).

Participants were classified as either low- or high-PRVS susceptibility on the basis of two subjective measures. First, a questionnaire was used to identify symptoms that participants had noticed prior to the testing session. Twenty questions were included, as detailed in a previous publication (Hollis and Allen, 2006). Each question received a score of 1 for a positive response and 0 for negative, giving a total score out of 20 for each participant. Scores of >4 were taken to indicate that a person is susceptible to visual stress and likely to experience symptoms. The second measure evaluated participants' direct subjective responses on viewing a high contrast pattern (Wilkins, 1995). The pattern was a horizontal grating having a square-wave luminance profile and Michelson contrast of 0.8. It was circular in outline and presented an overall diameter of 28° and spatial frequency of 3 cdeg^−1^ when viewed at a distance of 0.4 m. Participants viewed the grating and answered questions to identify the number of perceptual distortions they experienced. Scores of >3 were taken to indicate that a person is susceptible to PRVS and likely to experience symptoms.

**Figure 1:**
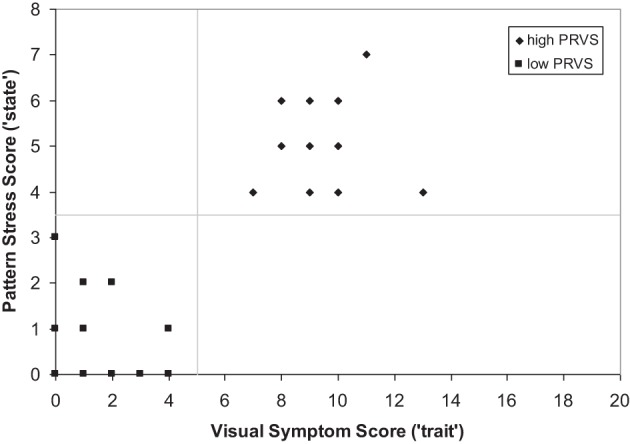
**Classification of low- and high-PRVS by reported visual symptoms and subjective response to pattern**.

Each participant was classified as exhibiting either low- or high-PRVS based upon responses to the visual symptoms questionnaire and pattern test. Scores above threshold were required on both measures; >4 for the visual symptoms questionnaire, >3 for pattern. Groups were formed from the first 14 participants who conformed to the classification criteria for each category. Those assigned to the low-PRVS group gave scores ranging from 0 to 4 (mean = 1.64, *sd* = 1.34) for the visual symptoms questionnaire, and from 0 to 3 (mean = 0.86, *sd* = 1.03) for pattern stress evaluation. Those assigned to the high-PRVS group gave scores ranging from 7 to 11 (mean = 9.07, *sd* = 1.69) for the visual symptoms questionnaire, and from 4 to 7 (mean = 4.93, *sd* = 1.00) for pattern glare evaluation. It is notable that low- and high-PRVS groups were well separated by both visual symptom scores [*t*_(26)_ = 12.92, *p* < 0.001] and pattern evaluation [*t*_(26)_ = 10.64, *p* < 0.001], and scores on the two measures were highly correlated (Spearman *R* = 0.78, *t* = 6.34, *p* < 0.001). The consequence of this is that the classifications given by the two measures are in perfect agreement (Figure 1).

One aim in group formation was to achieve a similarity in age distributions, as it is recognized that age affects the speed of lexical decisions. Hence, the groups had mean ages of 36.6 years (high-PRVS) and 39.3 years (low-PRVS), which were not significantly different (*p* = 0.524). We made no attempt to match the groups on IQ, which appears to be only weakly associated with lexical decision speed (e.g., Ratcliff et al., 2010).

### Design

The single experiment involved comparing the performance of two groups of participants on a visual lexical decision (VLD) task. The groups comprised individuals classified as having either low or high susceptibility to pattern-related visual stress (low-PRVS and high-PRVS), and performance was measured for lexical decisions on both word and non-word stimuli. Therefore, a 2-factor mixed design was employed, having 1 between-groups and 1 within-groups factor. The between-groups factor had 2 levels; low-PRVS and high-PRVS susceptibility. The within-groups stimulus factor also had 2 levels; word and non-word. Details of these are given in the following section. Dependent variables (performance measures) were the lexical decision response time in milliseconds and accuracy, recorded as percentage of correct responses. Statistical analysis was carried out using Statistica v8 (Statsoft Inc.).

### Stimuli and procedure

The set of experimental stimuli for the visual lexical decision task contained 96 items. These were presented in two sets of 48 items, each comprising 24 *words* (e.g., CASTLE) and 24 pronounceable *non-words* (e.g., HOLSE). Stimulus items are listed in Table 1. Non-words were selected …. All stimulus items had a length of between 4 and 6 characters (word average 4.8 characters, non-word average 4.9 characters). The average word frequency (Kucera-Francis) was 30. Words were selected to provide a heterogeneous stimulus set covering a wide range of occurrence frequencies, as is typically the case in everyday text. For the same reason, we made no attempt to constrain the orthographic characteristics of either words or non-words. A summary of these characteristics (length, word frequency, orthographic neighborhood size and frequency) is given in Supplementary Material; all measures were obtained using N-Watch (Davis, 2005).

**Table 1:** **Lists of word and non-word stimuli used in the study**.

**Set 1**		**Set 2**	
**Word**	**Non-word**	**Word**	**Non-word**
Cruel	Decey	Snake	Subel
Pour	Brant	Wind	Xale
Break	Pipso	Mist	Seent
Dose	Boret	Hitch	Nukel
Boar	Tane	Seep	Shair
Pipe	Brab	Brief	Sabem
Brain	Glave	Vent	Midlem
Daze	Prain	Throat	Rarks
Tablet	Wull	Click	Fillut
Grace	Chack	Jeeps	Jased
Surge	Tordl	Snail	Miest
Olive	Saereb	Tank	Liete
Sting	Blanet	Mouse	Saxeb
Truck	Glime	Island	Rudel
Belt	Jatde	Castle	Cacke
Riding	Filt	Knife	Wulst
Vase	Golk	Yacht	Rinem
Bear	Midel	Threat	Leven
Monk	Staem	Clerk	Colten
Steak	Shaty	Soot	Brabe
Sieve	Nucke	Chest	Tanon
Bowl	Seret	Toll	Ungle
Touch	Zalen	Pint	Pilep
Gross	Eubel	Broad	Raint

The software for this study was written using Superlab 1.68 (Cedrus Corporation). The experiment was run on an Apple Macintosh 540 c with an LCD display measuring 19.0 × 14.5 cm. The room was illuminated with mains voltage (50 Hz) fluorescent lighting (“daylight” CCT 6500 K) providing an illuminance of 300 lux on the horizontal working plane and 200 lux on the task display. The display was positioned so as to prevent reflections of room luminaires being visible to participants in the experiment. Stimuli were presented at the center of the display screen in Arial upper-case 12 pt bold font.

Participants were positioned at 60 cm from the screen and asked to decide as quickly and as accurately as possible whether each stimulus presentation was a word (Yes/No). Responses were given by key press. Each participant received two sets of 48 trials. The order of the set presentation and the order of stimuli within sets were randomized. Before the experimental data were obtained, all participants took part in a practice session involving a separate set of 8 stimulus items (4 words and 4 non-words). Each trial was initiated by a fixation cross for 500 ms followed by presentation of the stimulus item. The participant's response terminated the screen display, and the inter-stimulus interval was 2 s.

## Results

Analysis was conducted using 2-factor between-within ANOVA in keeping with the experimental design. Response Time (msec) and accuracy (% correct) data were analyzed separately. Response Time data were obtained from correct responses only and were trimmed using the procedure and criteria of Van Selst and Jolicoeur (1994). Accuracy data were transformed to improve normality prior to ANOVA analysis (Zar, 1996). A conventional criterion for statistical significance of α = 0.05 was adopted for all comparisons. Given that age is known to be associated with increased response times on lexical decision and other tasks, (Ratcliff et al., 2010), we considered using ANCOVA with participant AGE as a covariate. However, a pre-requisite for covariance analysis is the assumption of a linear relationship between covariate and dependent variable(s), and our data did not meet this criterion. Correlation coefficients (Pearson) between AGE and the dependent variables, in both low-PRVS and high-PRVS groups, ranged from 0.159 to 0.269 and none was statistically significant. For this reason we did not include AGE as a covariate in our analysis.

**Figure 2:**
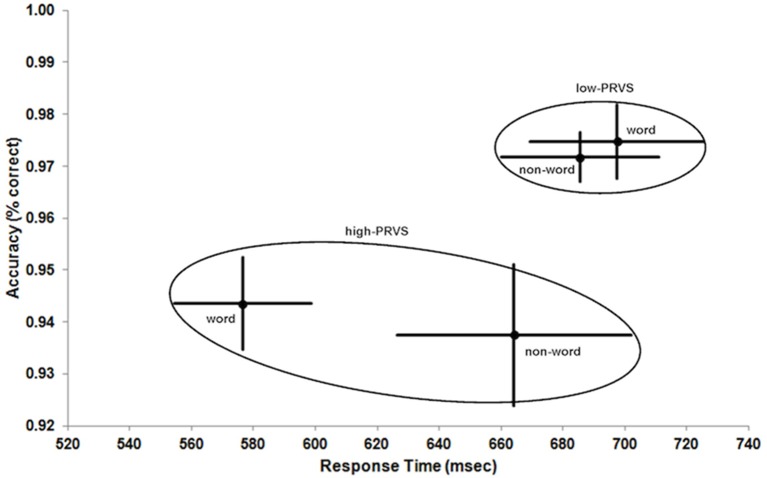
**Response Time vs. Accuracy of lexical decision for low- and high-PRVS (ellipses) on word and non-word stimuli**. Data points and bars indicate mean ± 1 standard error.

Figure 2 shows the results for the different group (low- and high-PRVS) and stimulus (word and non-word) conditions in speed-accuracy space. The data ranges (means ± 1 standard error) for each group are enclosed by ellipses to aid visualization of differences between and within groups.

The striking difference between the groups (Figure 2 and Table 2) is the lower accuracy of high-PRVS participants, and this effect is highly significant [low-PRVS = 97.3, high-PRVS = 94.1, *F*_(1, 26)_ = 16.010, *p* < 0.001, η^2^_*p*_ = 0.381]. Analysis of accuracy shows no significant effect of the stimulus type (word/non-word) and no significant interaction between stimulus and group. Response Time analysis shows that high-PRVS participants respond more quickly, though this effect just fails to reach significance [low-PRVS = 691.5, high-PRVS = 620.3, *F*_(1, 26)_ = 3.390, *p* = 0.077, η^2^_*p*_ = 0.115]. There is however a significant effect of stimulus type [*F*_(1, 26)_ = 7.963, *p* = 0.009, η^2^_*p*_ = 0.234], and significant interaction between stimulus and group [*F*_(1, 26)_ = 13.772, *p* < 0.001, η^2^_*p*_ = 0.346]. Overall, the pattern of speed-accuracy trade-off here is that those with high-PRVS favor speed at the expense of accuracy.

**Table 2:** **The full ANOVA for accuracy and response time data**.

	**Repeated measures ANOVA with effect sizes and powers**						
**Effect**	**SS**	**df**	**MS**	***F***	***p***	**η^2^*_p_***	**Observed power (α = 0.05)**
**ACCURACY**							
Group	150.0	1	150.0	16.01	0.00047	0.38110	0.970
Error	243.7	26	9.4				
Stimulus	2.8	1	2.8	0.20	0.65978	0.00757	0.071
Stim × Grp	0.3	1	0.3	0.02	0.88314	0.00085	0.052
Error	365.8	26	14.1				
**RESPONSE TIME**							
Group	70,858	1	70,858	3.390	0.07703	0.11535	0.426
Error	543,443	26	20,902				
Stimulus	20,064	1	20,064	7.963	0.00903	0.23447	0.775
Stim × Grp	34,701	1	34,701	13.772	0.00099	0.34628	0.946
Error	65,509	26	2520				

Within the low-PRVS group we find no significant difference in the responses to words and non-words, either in terms of speed [word = 697.4, non-word = 685.5, *F*_(1, 26)_ = 0.395, *p* = 0.535, η^2^_*p*_ = 0.015] or accuracy [word = 97.5, non-word = 97.2, *F*_(1, 26)_ = 0.044, *p* = 0.835, η^2^_*p*_ = 0.002]. Within the high-PRVS group there is no significant difference in the accuracy of responses to word and non-word stimuli [word = 94.4, non-word = 93.8, *F*_(1, 26)_ = 0.176, *p* = 0.678, η^2^_*p*_ = 0.007], but the response time difference is highly significant [word = 576.5, non-word = 664.1, *F*_(1, 26)_ = 21.340, *p* < 0.001, η^2^_*p*_ = 0.450].

**Figure 3:**
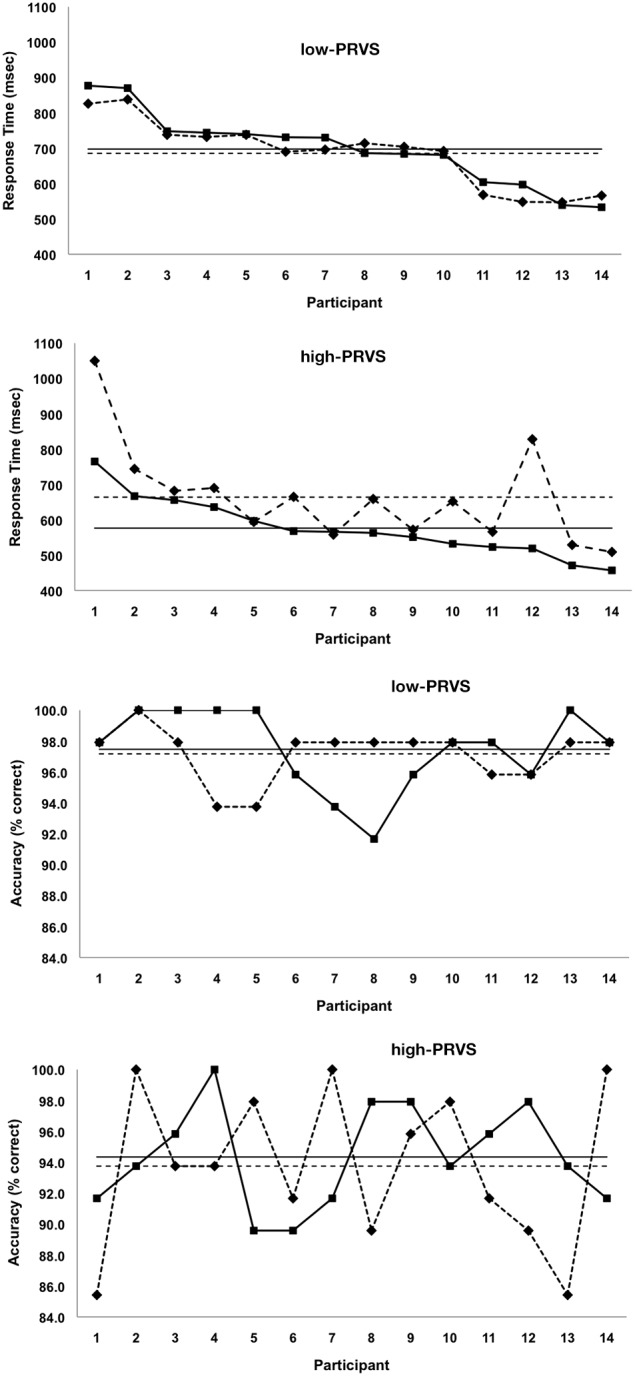
**Lexical decision response times and accuracies of individual participants, each group ordered 1 to 14 according to their highest to lowest response times for word stimuli**.

Additional insight into within and between-groups response differences is gained by examining plots of responses from individual participants (Figure 3).

In the panels of Figures 3A,B individual participants in low-PRVS and high-PRVS groups, respectively, are ordered from 1 to 14 according to their highest to lowest response times for word stimuli. Data points for words are joined by solid lines and those for non-words by dashed lines, while solid and dashed horizontal lines indicate mean response times for words and non-words respectively. Figure 3A shows that some individuals with low-PRVS respond more quickly to word stimuli while others respond more quickly to non-words. In every case the response time difference between words and non-words is small and, hence, the overall mean difference is not statistically significant. Figure 3B on the other hand shows that individual response times for non-words in the high-PRVS group are never lower but almost always higher than those for words. Although the magnitude of these individual differences varies substantially, the overall effect is of a significantly slower response to non-word stimuli.

Figures 3C,D show the corresponding plots of individual response accuracy for low-PRVS and high-PRVS respectively. Here the participants are ordered 1–14 as in Figures 3A,B, that is by the magnitude (highest to lowest) of their response times to word stimuli; accuracies for words are joined by solid lines and those for non-words by dashed lines, while solid and dashed horizontal lines indicate mean accuracies for words and non-words respectively. We see that there is no indication that response accuracy in either group is systematically better or poorer for non-words. Hence the overall effect, in both low- and high-PRVS, is that words and non-word accuracy is not significantly different, though the accuracy scores in high-PRVS participants are much variable than in those with low-PRVS.

## Discussion

The aim of this study was to explore whether performance on a visual lexical decision task differed between groups of adults having low and high susceptibility to pattern-related visual stress (PRVS). The hypothesis that high-PRVS participants may make lexical decisions with less accuracy is supported (Figure 2, *p* < 0.001, η^2^_*p*_ = 0.381). The hypothesis that high-PRVS participants may respond more slowly is not supported. On the contrary, lexical decision responses in high-PRVS are generally faster and, although the overall effect does not reach significance (*p* = 0.077, η^2^_*p*_ = 0.115), the pattern is of faster responses at the expense of accuracy in high-PRVS, compared with better accuracy and slower responses in low-PRVS. Thus, noting that all participants were instructed to make lexical decisions “as quickly and accurately as possible,” the differences here are consistent with the interpretation that the two groups adopt different strategies in the trade-off between speed and accuracy of lexical decisions.

In addition to the differences in responses between-groups we also find a striking difference within groups, in that there is no difference in the response times to words and non-words in low-PRVS, but there is a very significant response time difference to words and non-words in high-PRVS. This shows that the overall (non-significant) faster response of high-PRVS participants is in fact a combination of a relatively slow response to non-words, no different to that in low-PRVS, with a much faster response to words than is obtained in low-PRVS. In other words, we note that low-PRVS participants in this VLD task show no lexicality effect (*p* = 0.535, η^2^_*p*_ = 0.015), whereas those with high-PRVS show a highly significant lexicality effect (*p* < 0.001, η^2^_*p*_ = 0.450).

These results, showing faster but less accurate lexical decisions for words in high-PRVS, along with a selective lexicality effect, have not been demonstrated previously. They provide the first experimental indication of an association between sensory visual stress and the cognitive word-recognition aspect of reading. If these results are typical of individuals affected by pattern-related visual stress, then we have some evidence for how this condition may be implicated in poor reading performance. The overall effect could manifest either as reduced accuracy of word recognition, as in this experiment, or more generally as reduced reading speed in situations where there is a need to maintain accuracy in order to extract meaning from the text.

The faster response times for words in high-PRVS may be the effect of behavioral and/or physiological factors. A behavioral interpretation is that this may reflect an aversive response in that individuals with high-PRVS susceptibility, who commonly experience more visual discomfort and/or disturbance (Wilkins et al., 2004), may seek to avoid attending to the high contrast stimuli for longer than necessary and so favor speed of response over accuracy. Physiologically, reduced response times in high-PRVS should be consistent with the effects of cortical hyperexcitability, which has been proposed as a general explanation for PRVS (Wilkins et al., 1984) and has also been implicated in the heightened photosensitivity that occurs in some forms of migraine (Huang et al., 2003).

The lexicality effect, a difference in speed of response to word and non-word stimuli, is a typical pattern in the results of many lexical decision experiments. Generally, lexical decisions are expected to proceed at varying rates because more exhaustive search of the mental lexicon will be required when the stimulus is not immediately recognizable as a word (Forster and Bednall, 1976). Absence of a lexicality effect with low-PRVS participants in our experiment implies that these individuals do not need to invoke significantly different cognitive strategies for words and non-words; that is, the two types of letter string used in this experiment appear sufficiently far apart on the “lexical dimension line” that discriminating between them may be readily achieved through visual processing alone (Barca and Pezzulo, 2012). If this is the case then, by further implication, the strong lexicality effect observed in high-PRVS might be attributable to that condition interfering with normal visual processing of stimuli, such that the word and non-word stimuli are not immediately recognized as lexically distinct, and therefore the non-word stimuli must be processed for longer in a more exhaustive search of the lexicon.

Consider the situation if high-PRVS participants were to be required to perform to the same levels of accuracy as low-PRVS. In this case a simplistic approach, assuming reciprocation of response time and accuracy, would be to imagine moving the high-PRVS response-accuracy data point for words (Figure 2) along a linear trajectory until it equals that for low-PRVS. Here, responses times and accuracies for high-PRVS words, low-PRVS words and low-PRVS non-words will not be significantly different. Then, moving the high-PRVS non-word data point along a parallel trajectory will position it at the same higher level of accuracy as all the other data points but now its response time will be significantly longer than all other conditions. This approach puts the interpretation of these results into a different perspective, as now the emphasis is not on the faster responses of high-PRVS participants but on occurrence of an apparent relative non-word response time deficit in high-PRVS compared with low-PRVS.

As discussed previously, differences in *naming* of word and non-word stimuli (lexicality effects) may be interpreted in terms of the need for a slower phonological pathway for non-word recognition and naming (Coltheart et al., 2001). Similarly, a common interpretation of situations in which some individuals exhibit a non-word deficit not present in others is that this reflects a deficit in phonological processing. Indeed, occurrence of a phonological processing deficit associated with poor non-word reading is now well established as a defining characteristic of developmental dyslexia (Rack et al., 1992; Ijzendoorn and Bus, 1994; Herrmann et al., 2006). Certainly when individuals are required to sound out pronounceable non-words in a naming task then explicit orthographic-to-phonological conversion must be required, and those with poor phonological processing skills will exhibit a non-word naming deficit. However, for lexical decisions, requiring only classification of a stimulus as word or non-word, we do not expect the task to require phonological processing. In this context, therefore, it is much more likely that the apparent non-word deficit in high-PRVS is the result of a visual processing difference.

It seems that the aspect of visual processing most likely to be affected in this case is spatial attention. Auclair and Siéroff (2002), for example, reported results confirming an early role of visual attention in the process of word and non-word identification and concluded that “the lexical status of a letter string can directly influence the distribution of attention before the identification process is completely achieved,” while Kinsey et al. (2004) conclude that “the better one's attentional processing ability, the better one is at non-word reading.” Facoetti et al. (2006) also report results suggesting that focused visuo-spatial attention may be crucial for non-word decoding. More broadly, the role of visual attentional processing in word recognition is now widely recognized. For example, Lobier et al. (2012) report fMRI results showing activation in the posterior parietal cortex (PPC) suggesting that pre-orthographic attentional processing plays an important role in word recognition. Similarly, Pammer et al. (2006) report MEG results showing PPC activation in word recognition, suggesting that attentional processing in the dorsal pathway is important in visual word recognition. Going further still, Vidyasagar and Pammer (2009) propose that dyslexia is in fact attributable to a deficit in visuo-spatial attention, not in phonological processing.

The results we report here can provide no direct evidence of whether visual attentional processes generally are affected in those with high-PRVS susceptibility, and we are not aware of any other reports to this effect. However, given that our results are the first to evidence differences in word recognition performance in high-PRVS, we hope that the speculation within this discussion may provide a framework to support further work.

## Limitations and concluding remarks

We have presented this study as a preliminary investigation due to its limitations. First, one reviewer commented that the group sample size is “exaggeratedly low.” We accept that it would be desirable to increase this in future studies, though we note that our analysis here (Table 2) shows high statistical power on significant effects in spite of the modest sample size. Second, and a particularly important limitation in the context of this work, is the fact that original data noting the responses to individual stimulus items were lost. This regrettably occurred due to the original raw data and trimmed mean data being held in separate files on different computers, one of which (holding the raw data) suffered damage beyond repair. Always back up your data! This lack of original data on responses per item severely limits the analysis that can be carried out; we are unable to present statistics “by-subject” and “by-item” that might provide further insight into response patterns in the two groups of interest and support generalization of the results. Likewise, although we deliberately selected stimuli in a naturalistic fashion with highly heterogeneous distributions of word frequency and neighborhood characteristics, it would have been of interest to examine whether response patterns in the low- and high-PRVS groups showed any association with such characteristics. Third, the premise of the study is that word recognition may be affected by pattern-related visual stress but, as discussed in the introduction, views on the concept, existence and effects of visual stress vary and there is as yet no robust method for definition and measurement of the condition that does not rely mainly (if not entirely) upon subjective symptom reports. Notwithstanding all of these limitations, however, we believe that our findings are worthy of report. Although the low- and high-PRVS groups were defined by reported symptoms, the difference in magnitude of these was very marked and participants could be sharply discriminated by these criteria. Furthermore, in the analysis of correct response time, the definitive measure of lexical decision performance, the magnitude of the interaction between stimulus type (word/non-word) and participant group is highly significant over a very heterogeneous stimulus set and, as far as we are aware, no such effect has been reported previously. We hope that this will provide a spur for other researchers to explore this topic and provide a starting point for more extensive and rigorous investigations.

### Conflict of interest statement

The authors declare that the research was conducted in the absence of any commercial or financial relationships that could be construed as a potential conflict of interest.
