# Supplementary material for: Lexical decisions in adults with low and high susceptibility to pattern-related visual stress: a preliminary investigation
Source: Front Psychol. 2015 Apr 14;6:449. doi: 10.3389/fpsyg.2015.00449 (PMC4396132; doi:10.3389/fpsyg.2015.00449)
Supplement: Supplementary file 1 [file DataSheet1.DOCX]

Appendix

|  |  | **WORD FREQUENCY** | | | **NEIGHBOURHOOD** | |
| --- | --- | --- | --- | --- | --- | --- |
| **ITEM** | **LENGTH** | **CELEX** | **KF** | **BNC** | **Size N** | **Mean Freq.** |
| bear | 4 | 70.39 | 58 | 58.65 | 18 | 78.99 |
| belt | 4 | 21.17 | 30 | 21.78 | 10 | 90.63 |
| boar | 4 | 2.01 | 1 | 4.11 | 5 | 28.25 |
| bowl | 4 | 29.27 | 23 | 24.73 | 7 | 4.58 |
| brain | 5 | 69.22 | 47 | 50.07 | 5 | 24.9 |
| break | 5 | 105.03 | 93 | 93.08 | 6 | 16.52 |
| brief | 5 | 46.15 | 74 | 56.54 | 1 | 15.53 |
| broad | 5 | 42.79 | 89 | 55.72 | 2 | 38.97 |
| castle | 6 | 59.22 | 8 | 60.26 | 1 | 31.73 |
| chest | 5 | 43.46 | 56 | 39.39 | 3 | 8.46 |
| clerk | 5 | 23.52 | 34 | 19.61 | 0 | 0 |
| click | 5 | 7.71 | 2 | 6.03 | 8 | 7.03 |
| cruel | 5 | 27.04 | 15 | 14.39 | 3 | 0.75 |
| daze | 4 | 2.23 | 0 | 1.13 | 10 | 14.13 |
| dose | 4 | 6.82 | 12 | 17.57 | 12 | 58.64 |
| grace | 5 | 25.42 | 40 | 25.24 | 7 | 11.83 |
| gross | 5 | 22.01 | 66 | 25.95 | 5 | 35.26 |
| hitch | 5 | 3.69 | 5 | 3.75 | 6 | 10.94 |
| island | 6 | 67.54 | 167 | 71.79 | 1 | 12.07 |
| jeeps | 5 | 2.01 | 0 | 1.37 | 4 | 8.59 |
| knife | 5 | 36.93 | 80 | 27.94 | 0 | 0 |
| mist | 4 | 13.63 | 14 | 12.36 | 8 | 306.19 |
| monk | 4 | 3.91 | 16 | 6.86 | 3 | 3.97 |
| mouse | 5 | 8.16 | 10 | 19.06 | 6 | 93.92 |
| olive | 5 | 11.17 | 7 | 9.75 | 1 | 63.41 |
| pint | 4 | 10.34 | 14 | 11.62 | 11 | 9.01 |
| pipe | 4 | 22.91 | 20 | 26.11 | 7 | 12.55 |
| pour | 4 | 24.64 | 9 | 13.02 | 8 | 297.9 |
| riding | 6 | 26.2 | 45 | 26.56 | 4 | 19.97 |
| seep | 4 | 1.06 | 2 | 1.1 | 13 | 99.81 |
| sieve | 5 | 2.46 | 1 | 1.93 | 1 | 8.32 |
| snail | 5 | 2.57 | 1 | 2.94 | 1 | 1.62 |
| snake | 5 | 14.58 | 46 | 8 | 4 | 10.01 |
| soot | 4 | 1.9 | 1 | 1.93 | 16 | 61.94 |
| steak | 5 | 8.16 | 10 | 4.19 | 5 | 32.9 |
| sting | 5 | 5.25 | 5 | 6.15 | 6 | 7.09 |
| surge | 5 | 7.77 | 9 | 8.85 | 2 | 2.68 |
| tablet | 6 | 2.68 | 3 | 3.36 | 2 | 16.03 |
| tank | 4 | 20.34 | 13 | 34.99 | 12 | 42.2 |
| threat | 6 | 61.4 | 42 | 61 | 2 | 27.04 |
| throat | 6 | 43.02 | 51 | 33.96 | 1 | 61.4 |
| toll | 4 | 7.21 | 25 | 8.81 | 12 | 96.51 |
| touch | 5 | 95.81 | 87 | 68.13 | 5 | 11.79 |
| truck | 5 | 24.8 | 60 | 12.37 | 4 | 21.37 |
| vase | 4 | 3.97 | 4 | 5.09 | 6 | 92.98 |
| vent | 4 | 3.13 | 10 | 3.75 | 10 | 126.67 |
| wind | 4 | 110.89 | 70 | 79.44 | 12 | 128.01 |
| yacht | 5 | 4.64 | 4 | 11.15 | 0 | 0 |
|  |  |  |  |  |  |  |
| mean | 4.75 | 26.17 | 30.81 | 24.20 | 5.75 | 44.86 |
| stdev | 0.67 | 28.42 | 34.36 | 24.10 | 4.42 | 64.65 |

|  |  | **WORD FREQUENCY** | | | **NEIGHBOURHOOD** | |
| --- | --- | --- | --- | --- | --- | --- |
| **ITEM** | **LENGTH** | **CELEX** | **KF** | **BNC** | **Size N** | **Mean Freq.** |
| blanet | 6 | 0 | 0 | 0 | 1 | 25.03 |
| boret | 5 | 0 | 0 | 0 | 3 | 9.57 |
| brab | 4 | 0 | 0 | 0 | 9 | 3.58 |
| brabe | 5 | 0 | 0 | 0 | 4 | 8.14 |
| brant | 5 | 0 | 0 | 0 | 3 | 15.51 |
| cacke | 5 | 0 | 0 | 0 | 1 | 0.84 |
| chack | 5 | 0 | 0 | 0 | 8 | 13.46 |
| colten | 6 | 0 | 0 | 0 | 1 | 3.13 |
| decey | 5 | 0 | 0 | 0 | 3 | 4.19 |
| eubel | 5 | 0 | 0 | 0 | 0 | 0 |
| fillut | 6 | 0 | 0 | 0 | 1 | 1.45 |
| filt | 4 | 0 | 0 | 0 | 13 | 49.96 |
| glave | 5 | 0 | 0 | 0 | 6 | 10.51 |
| glime | 5 | 0 | 0 | 0 | 3 | 1.66 |
| golk | 4 | 0 | 0 | 0 | 4 | 34.57 |
| jased | 5 | 0 | 0 | 0 | 3 | 3.57 |
| jatde | 5 | 0 | 0 | 0 | 0 | 0 |
| leven | 5 | 0 | 0 | 0 | 5 | 64.45 |
| liete | 5 | 0 | 0 | 0 | 1 | 0.39 |
| midel | 5 | 0 | 0 | 0 | 1 | 55.47 |
| midlem | 6 | 0 | 0 | 0 | 0 | 0 |
| miest | 5 | 0 | 0 | 0 | 1 | 13.24 |
| nucke | 5 | 0 | 0 | 0 | 0 | 0 |
| nukel | 5 | 0 | 0 | 0 | 0 | 0 |
| pilep | 5 | 0 | 0 | 0 | 2 | 10.81 |
| pipso | 5 | 0 | 0 | 0 | 0 | 0 |
| prain | 5 | 0 | 0 | 0 | 6 | 41.04 |
| raint | 5 | 0 | 0 | 0 | 6 | 16.68 |
| rarks | 5 | 0 | 0 | 0 | 6 | 11.96 |
| rinem | 5 | 0 | 0 | 0 | 0 | 0 |
| rudel | 5 | 0 | 0 | 0 | 1 | 0.56 |
| sabem | 5 | 0 | 0 | 0 | 0 | 0 |
| saereb | 6 | 0 | 0 | 0 | 0 | 0 |
| saxeb | 5 | 0 | 0 | 0 | 0 | 0 |
| seent | 5 | 0 | 0 | 0 | 2 | 77.68 |
| seret | 5 | 0 | 0 | 0 | 1 | 1.79 |
| shair | 5 | 0 | 0 | 0 | 2 | 53.83 |
| shaty | 5 | 0 | 0 | 0 | 2 | 4.05 |
| staem | 5 | 0 | 0 | 0 | 0 | 0 |
| subel | 5 | 0 | 0 | 0 | 0 | 0 |
| tane | 4 | 0 | 0 | 0 | 18 | 53.68 |
| tanon | 5 | 0 | 0 | 0 | 1 | 4.19 |
| tordl | 5 | 0 | 0 | 0 | 0 | 0 |
| ungle | 5 | 0 | 0 | 0 | 2 | 41.56 |
| wull | 4 | 0 | 0 | 0 | 13 | 309.67 |
| wulst | 5 | 0 | 0 | 0 | 0 | 0 |
| xale | 4 | 0 | 0 | 0 | 10 | 21.2 |
| zalen | 5 | 0 | 0 | 0 | 0 | 0 |
|  |  |  |  |  |  |  |
| mean | 4.98 | 0.00 | 0.00 | 0.00 | 2.98 | 20.15 |
| stdev | 0.48 | 0.00 | 0.00 | 0.00 | 4.01 | 47.29 |
